# Novel insights into the origin and development of CNS macrophage subsets

**DOI:** 10.1002/ctm2.1096

**Published:** 2022-11-06

**Authors:** Takahiro Masuda, Lukas Amann, Marco Prinz

**Affiliations:** ^1^ Department of Molecular and System Pharmacology Graduate School of Pharmaceutical Sciences Kyushu University Fukuoka Japan; ^2^ Institute of Neuropathology, Faculty of Medicine University of Freiburg Freiburg Germany; ^3^ Center for Basics in NeuroModulation (NeuroModulBasics), Faculty of Medicine University of Freiburg Freiburg Germany; ^4^ Signalling Research Centres BIOSS and CIBSS University of Freiburg Freiburg Germany

**Keywords:** CNS, development, macrophage, microglia, origin

## Abstract

The central nervous system (CNS) hosts a variety of immune cells, including two distinct macrophage populations: microglia are found in the parenchyma, whereas CNS‐associated macrophages (CAMs) cover the CNS interfaces, such as the perivascular spaces, the meninges and the choroid plexus. Recent studies have given novel insights into the nature of CAMs as compared to microglia. In this mini‐review, we summarise the current knowledge about the ontogenetic relationship and the underlying mechanism for the establishment of CNS macrophages during development.

## Graphical abstract

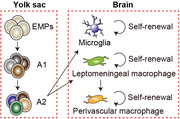

## 1 ANATOMICALLY AND TRANSCRIPTIONALLY DISTINCT MACROPHAGE POPULATION IN THE CENTRAL NERVOUS SYSTEM

**FIGURE 1:**
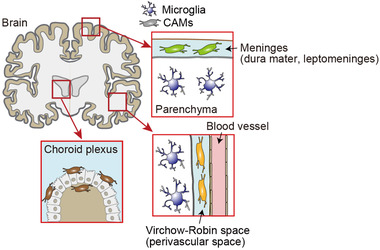
Anatomically distinct subsets of central nervous system (CNS) macrophages. Microglia reside in the parenchyma of the CNS, whereas non‐parenchymal CNS‐associated macrophages (CAMs) are localised at the interfaces, such as the meninges (dura mater and leptomeninges), the perivascular spaces (Virchow–Robin spaces) and the choroid plexus.

Microglia have long been regarded as the primary immune cell in the central nervous system (CNS) including brain and spinal cord, and they are shown to play various functions during development and adulthood under both homeostatic and disease conditions.^1^, ^2^ A current hot topic in the research field is their cellular heterogeneity, which recently just started to be deeply investigated with the help of novel single‐cell techniques, such as single‐cell RNA sequencing (scRNA‐seq), and already gave a big impact through the discovery of microglia subsets or substates during development and in disease.^3^, ^4^, ^5^, ^6^, ^7^ On the other hand, besides microglia, there are other anatomically distinct macrophage subsets in the CNS, known as CNS‐associated macrophages (CAMs) that are localised at the non‐parenchymal CNS interfaces, such as the perivascular space (Virchow–Robin space), the meninges that consist of dura matter and leptomeninges and the choroid plexus^8^, ^9^ (Figure 1). Importantly, microglia and CAMs share several cell markers including the ionised calcium‐binding adaptor molecule 1 (IBA1), the fractalkine receptor (*Cx3cr1*), or the colony‐stimulating factor 1 receptor (*Csf1r*), making it difficult to clearly segregate the function of CAMs from that of microglia.^10^ However, recent scRNA‐seq data have shown the distinct transcriptomic features of microglia and CAMs. These include high gene expression of mannose receptor C‐type 1 (*Mrc1*), lymphatic vessel endothelial hyaluronan receptor 1 (*Lyve1*), membrane spanning 4‐domains a7 (*Ms4a7*), platelet factor 4 (*Pf4*) and *Cd163* in CAMs, whereas microglia can be characterised by high expression of beta‐hexosaminidase subunit beta (*Hexb*), P2Y purinergic receptor 12 (*P2ry12*), transmembrane protein 119 (*Tmem119*) and solute carrier family 2, facilitated glucose transporter member 5 (*Slc2a5*),^8^, ^11^, ^12^ suggestive of potentially diverse functions during physiology and pathophysiology.

## 2 GENETIC TOOLS TO STUDY CAMS

To explore the nature of CAMs, including their ontogeny and the specific functions during homeostatic development and disease, genetic tools that target CAMs are vital. The previously generated transgenic mouse lines targeting CNS myeloid cells, such as *Cx3cr1^CreER^* or *Csf1r^Mer‐iCre‐Mer^* lines, are in many cases useful for studying CAMs, but unfortunately unable to discriminate CAMs from other CNS myeloid cells like microglia. On the other hand, there are now several transgenic mice available for specifically studying the biology of CAMs. For instance, *Lyve1^EGFP/cre^* mice, in which expression of Cre recombinase with enhanced green fluorescent protein (EGFP) in these knock‐in mice is driven by the *Lyve1* promoter,^13^ or *Pf4^iCre^* mice in which an improved Cre recombinase (iCre) is induced under the control of the Pf4 promoter,^14^, ^15^ allow cell type‐specific gene targeting for CAMs. In addition, to study the definition and dissection of the precise ontogeny and the specific functions of CAMs, we have recently developed a novel mouse line by applying CRISPR/Cas9 genome editing, which enables to target CAMs in time‐controlled and cell type‐specific manners (*Mrc1^CreERT2^*) without affecting other CNS cells or circulating blood cells.^16^ In combination with recently developed tools to specifically target microglia (e.g., *Hexb^CreERT2^* ^12^, *P2ry12^CreERT2^* ^15^ and *Tmem119^CreERT2^* ^17^), our newly developed line would provide a valuable option to segregate the functions of CAMs from those of microglia during development, homeostasis and diseases of the CNS.

## 3 ORIGIN AND MAINTENANCE OF TISSUE RESIDENT MACROPHAGES IN THE CENTRAL NERVOUS SYSTEM

**FIGURE 2:**
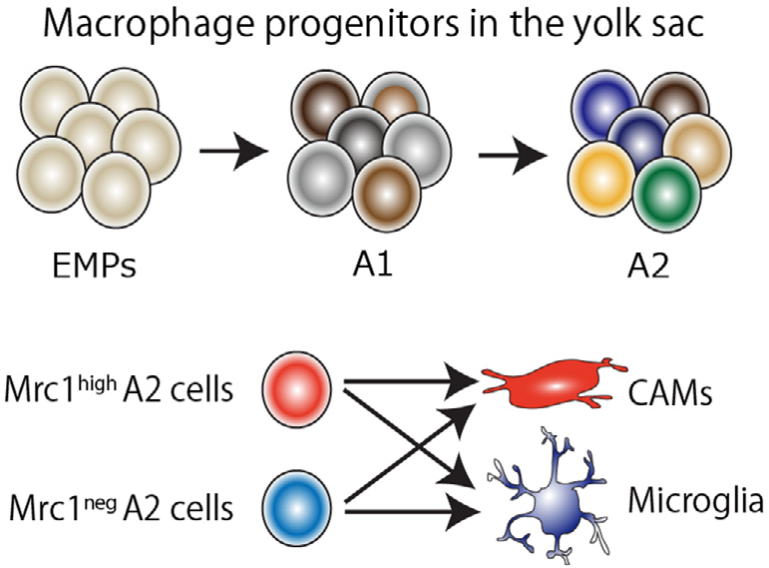
Central nervous system (CNS)‐associated macrophages (CAMs) and microglia share a common macrophage progenitor in the yolk sac. During embryogenesis, erythro‐myeloid progenitors (EMPs) arise in the blood island of the extraembryonic yolk sac, which give rise to A2 pre‐macrophage progenitors via A1 immature progenitors. The A2 progenitors include the cells with high expression of *Mrc1* (Mrc1^high^) and Mrc1‐negative population (Mrc1^neg^), both of which can give rise either to microglia or CAMs in the CNS.

Similar to most of tissue resident macrophages, including microglia, CAMs are originally derived from prenatal progenitors that arise in the extra‐embryonic yolk sac blood island, which has been defined as erythro‐myeloid progenitor (EMPs), giving rise to immature A1 macrophage progenitors that further differentiate into A2 pre‐macrophage progenitors.^18^, ^19^ From embryonic day 9.5 (E9.5) on, first CAMs population can be observed surrounding the developing brain where meninges are being established during embryogenesis, as is the case for microglia in the parenchyma. However, whether microglia and CAMs share a common progenitor, or whether distinct pre‐committed precursors already exist in the yolk sac remained unknown. Utz et al. (2020) recently described two phenotypically and transcriptionally distinct macrophage populations, which can be distinguished by their expression of CD206 (CD206^–^ vs. CD206^+^) in the yolk sac and the brains from early embryogenesis to adulthood.^11^ In the light of such continuous appearance of CD206^+^ cells, they concluded that microglia and CAMs are separate populations already from the emergence of primitive macrophages in the yolk sac. Independently, we recently assessed the gene expression profile of A1 and A2 macrophage progenitors in the yolk sac at single‐cell resolution, and found the presence of nine transcriptionally distinct clusters, including two clusters that constitute the *Cx3cr1^hi^Ptprc^+^* matured population.^16^ Of note, one of these two clusters was characterised with high expression level of genes including *Mrc1* (encoding CD206), raising the possibility that the *Mrc1* ^+^ A2 macrophage progenitors may be the committed yolk sac progenitors for CAMs, in line with the idea by Utz et al.^11^ To confirm this, we adapted a fate‐mapping system with our novel *Mrc1^CreERT2^* mouse line in which a T2A‐CreERT2 cassette was inserted into the *Mrc1* locus, which had been crossed with *Rosa26* ^t^ *^dTomato^* ^/+^ (*R26^tdT^*), allowing to specifically and permanently label *Mrc1^+^* macrophage progenitors in the yolk sac. However, contrary to our expectation, analysis of the offspring revealed the presence of tdT‐expressing cells in both microglia and CAMs,^16^ indicating that CAMs and microglia share the common *Mrc1* ^+^ yolk sac progenitor and that the determination of cell fate occurs locally within the developing anatomical CNS niche (Figure 2), though it remains unknown if this concept is also applicable for macrophages in the choroid plexus.

## 4 TIMING AND MECHANISM FOR THE DISTRIBUTION OF CAM SUBSETS DURING DEVELOPMENT

**FIGURE 3:**
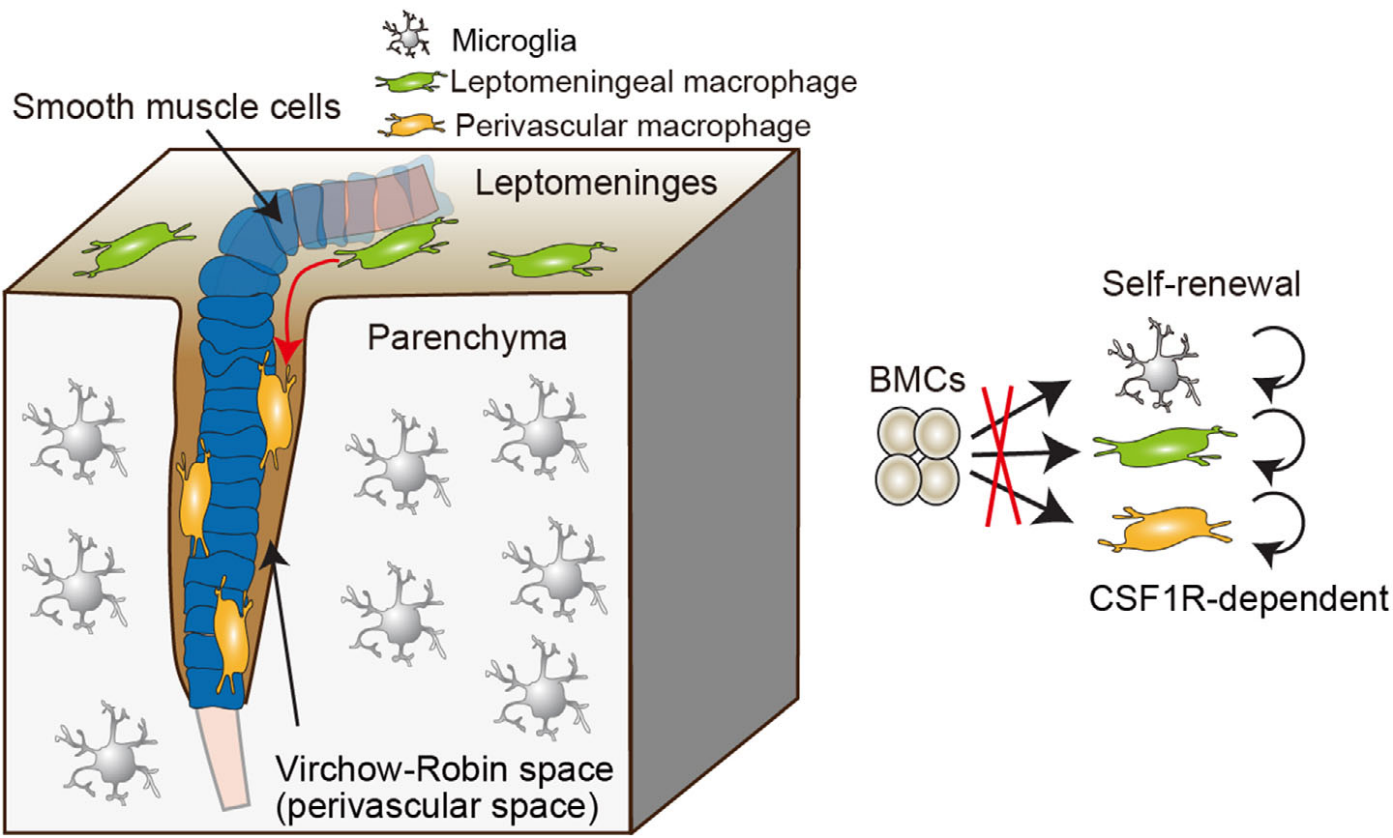
Perivascular macrophages require arterial vascular smooth muscle cells for their proper distribution from leptomeninges. After the Virchow–Robin spaces develop, the perivascular macrophages that originate from perinatal leptomeningeal macrophages are established in the spaces, which requires the presence of vascular smooth muscle cells. After being established in the CNS, microglia and perivascular and leptomeningeal macrophages are maintained by self‐renewal in a manner that depends on colony‐stimulating factor 1 receptor (CSF1R) signal, without any contribution of bone marrow‐derived cells (BMCs).

Both microglia and CAMs are known to be established prenatally and maintained whole life long with low self‐renewal capacity in a manner that depends on CSF1 receptor signal (Figure 3),^9^ with the exception of dura mater macrophages and stromal macrophages in the choroid plexus, which are continuously replaced with bone marrow‐derived cells under homeostatic condition.^8^ However, our recent in‐depth analysis revealed unexpected developmental kinetics of perivascular macrophages, which are distributed only after birth, along with the concomitant establishment of the Virchow–Robin space,^16^ sandwiched within two basal laminas (one from the endothelial cells or basement membranes of mural cells and the other from the astrocytic endfeet).^18^, ^20^ The similar developmental pattern of perivascular macrophages was observed also in human brain, suggesting an evolutionally conserved feature. Another striking characteristic of perivascular macrophages was their preferential distribution surrounding arteries and arterioles in mice and human, and in a sex‐independent manner^16^ (Figure 3). There was a minor population of perivascular macrophages colonising the veins and venules, but capillaries did not host perivascular macrophages at all. From where perivascular macrophages supply after birth? After excluding bone marrow‐derived cells and microglia as a potential contributor to the perivascular macrophages, our fate mapping analysis together with confetti system concluded that during early postnatal development, leptomeningeal macrophages continuously infiltrate into the perivascular space and expand by local proliferation^16^ (Figure 3). In other words, developing leptomeninges serve as an intermediate environmental niche for postnatal introduction of perivascular macrophages when the perivascular niche develops. To understand the underlying mechanism for the establishment of perivascular macrophages, a bulk RNA‐seq analysis of perivascular and leptomeningeal macrophages was performed, which uncovered an age‐dependent regulation in the expression levels of several integrin‐related genes including Talin‐1 (*Tln1*), a cytosolic adaptor protein that controls the activation of integrin‐mediated signaling pathways.^21^ In mice lacking *Tln1* in brain myeloid cells, including CAMs, the number of perivascular macrophages was robustly decreased, with no differences in the number of vascular branching points or parameters of vascular integrity.^16^ In addition, *Tln1* deficiency changed the morphology of perivascular macrophages to be roundish. Thus, perivascular macrophages require integrin signals for their proper distribution in the perivascular space.

The complex structure of brain vasculature consists of diverse cell types including astrocytes and mural cells, such as pericytes or vascular smooth muscle cells (VSMCs).^22^ Among those cell types are VSMCs that were found to be crucial for the proper distribution of perivascular macrophages during development, as Notch3‐deficient brains, in which arterial VSMCs are impaired and reduced,^23^ had less perivascular macrophages, although microglia and leptomeningeal macrophages were normal.^16^ Importantly, no apparent changes of the proximal perivascular spaces were detected, indicating that the spatial anatomical preconditions for proper perivascular macrophage seeding were unaltered by Notch3 deficiency. In contrast, a pronounced but incomplete arterial‐to‐venous shift in the transcriptomic profile of arterial VSMCs, as with lower expression of arterial genes and concomitantly increased levels of venous genes, was evident. Although the key molecules on or signals from arterial VSMCs that contribute to the distribution of perivascular macrophages have not been identified, the presence of arterial VSMCs regulated by Notch3 is crucial for perivascular macrophage development (Figure 3).

## 5 CONCLUDING REMARKS

Recent advances in research technology offer deep insights into the exciting features of CAMs, which undoubtedly help better understand how the CNS develops and functions properly. Nevertheless, there is still a long list of open questions that needs to be answered. Especially, the behaviour and functions of CAMs during normal CNS development, steady state and disease remain to be poorly defined, at least partially due to the lack of tools for specifically targeting CAMs, for which we recently developed a novel transgenic mouse line. Accordingly, whether CAMs are detrimental or beneficial for disease progression in both mice and humans remains elusive. Answering such critical questions together with profiling CAMs in healthy and diseased situations will open new avenues for the development of therapeutic targeting of CAMs.

## CONFLICT OF INTEREST

The authors declare they have no conflicts of interest.
